# Retraction: Therapeutic Potential of Date Palm Pollen for Testicular Dysfunction Induced by Thyroid Disorders in Male Rats

**DOI:** 10.1371/journal.pone.0348626

**Published:** 2026-05-05

**Authors:** The PLOS One Editors

After this article [1] was published, concerns were raised about Fig 5. Specifically,

- Several comet tails in Figs 5A and C-F appear similar to one another, both within individual panels and between different panels.
- All panels of Fig 5 present repeating cell grouping patterns, which appear more similar than would be expected from independent samples.
- When levels are adjusted, the background immediately surrounding cells in all panels of Fig 5 appear discontinuous with the adjacent background.

In light of the above concerns, which call into question the reliability and integrity of the published results, the *PLOS One* Editors retract this article.

MMN agreed with the retraction. AMEK did not agree with the retraction. WAH and SMR either did not respond directly or could not be reached.
